# Willingness of Healthcare Workers to Recommend or Receive a Third COVID-19 Vaccine Dose: A Cross-Sectional Study from Jordan

**DOI:** 10.3390/idr15020022

**Published:** 2023-04-06

**Authors:** Mohammad Abu Lubad, Munir A. Abu-Helalah, Israa F. Alahmad, Malak M. Al-Tamimi, Mohammad S. QawaQzeh, Ahlam M. Al-kharabsheh, Hamed Alzoubi, Ahmad H. Alnawafleh, Khalid A. Kheirallah

**Affiliations:** 1 Department of Microbiology and Pathology, Faculty of Medicine, Mutah University, Alkarak 61710, Jordan; 2 Department of Public Health, Faculty of Medicine, Mutah University, Alkarak 61710, Jordan; 3 Faculty of Medicine, Medical Students at Mutah University, Alkarak 61710, Jordan; 4 Department Obstetrics and Gynecology, Faculty of Medicine, Mutah University, Alkarak 61710, Jordan; 5 Board Member, Jordan Centre for Disease Control, Amman 11814, Jordan; 6 Department Adult Health Nursing, Faculty of Nursing, Mutah University, Alkarak 61710, Jordan; 7 Department of Public Health, Community Medicine, Jordan University of Science and Technology, Irbid 22110, Jordan

**Keywords:** cross-sectional study, public health problem, chronic disease, willingness, vaccine dose, World Health Organization, health care workers, COVID-19, health promotion program

## Abstract

Background: The availability of COVID-19 vaccines worldwide necessitates measuring healthcare workers’ (HCWs’) willingness to recommend or receive these vaccines. Therefore, we conducted a local study in Jordan to assess HCWs’ willingness to recommend or receive a third dose of a COVID-19 vaccine and the predictors of such a decision. A cross-sectional study investigated Jordanian HCWs’ willingness regarding a third dose of a COVID-19 vaccine using a self-administered online questionnaire through WhatsApp, a mobile phone application. A total of 300 HCWs participated in the current study. Of these HCWs, 65.3% were physicians, 25.3% were nurses, and 9.3% were pharmacists. HCWs’ overall willingness regarding a third vaccine dose was 68.4% (49.4% certainly and 19.0% probably), whereas the overall willingness of HCWs to recommend a third dose to their patients was 73.3% (49.0% certainly and 24.3% probably). Males had significantly higher willingness than females (82.1% vs. 60.1%, *p* < 0.05). Physicians reported more willingness than nurses and pharmacists. HCWs’ willingness was not significantly affected by direct contact with a patient infected with COVID-19 or by a personal history of COVID-19 infection. Only 31% of HCWs were certainly willing to recommend the vaccine to their patients with chronic diseases, and only 28% of the participants were certainly willing to recommend it to people aged 65 or older. HCWs’ willingness to receive a third dose of a COVID-19 vaccine is limited in Jordan. This has affected their certainty in recommending this vaccine to their patients or people older than 60. Decision-makers and health-promotion programs in Jordan should focus on addressing this public health problem.

## 1. Introduction

The World Health Organization (WHO) declared a pandemic in March 2020 associated with SARS-CoV-2 [1,2]. Since late 2020, several vaccines have been made available worldwide to assist in the global efforts to control this pandemic. The vaccination program to combat Coronavirus disease (COVID-19) in Jordan started in January 2021. Different vaccines have been used in Jordan, including Oxford University—AstraZeneca, Pfizer—BioNTeck (PB), Moderna, Gamaleya (Sputnike V), and SinoPharm (SP) [3].

Healthcare workers (HCWs) have been given priority for vaccination for COVID-19 to assist them in handling COVID-19 patients and other patients seeking medical advice during this pandemic. Many countries made vaccination compulsory for healthcare professionals, whereas others have not followed this approach [4].

The World Health Organization defines vaccine hesitancy as a “delay in acceptance or refusal of vaccines despite availability of vaccination services”, whereas vaccination unwillingness refers to “the refusal to be vaccinated”. COVID-19 vaccine unwillingness can be a major barrier to achieving sufficient vaccine coverage to contain the pandemic [5,6,7]. On the other hand, COVID-19 vaccination willingness among HCWs is defined as their willingness to receive a full dose of vaccination or take the first or second COVID-19 vaccine [8,9]. Recently, the emergence of new SARS-CoV-2 variants, including the B.1.617.2 (delta) and the B.1.1.529 (omicron) variants, has necessitated a third dose of the COVID-19 vaccine to decrease the associated morbidity and mortality rates [10,11]. The three-dose vaccine program could be helpful in reducing the risk of infection with COVID-19 and its variants. This can be achieved by inducing more neutralizing antibodies to overcome the decline in titer that has been observed over time after the second dose [12,13]. One of the key difficulties in promoting the uptake of the third dose in HCWs lies in ensuring good knowledge about the risks and benefits of the vaccination. Additionally, the uptake of the third dose amongst HCWs could affect their approach to promoting the third dose amongst their patients and the general population [5]. 

A study from the USA showed that only 7.9% (n = 107) of HCWs were hesitant to take the first or second dose of the vaccine. Vaccine-hesitant respondents were mainly younger HCWs (18–40 years) and HCWs with lower levels of educational attainment at secondary school. Among the vaccine-hesitant respondents, the acceptance of a hypothetical third dose was only 14.3% [14].

In Jordan, a previous study showed that only 45.6% of HCWs were willing to take the first dose of the COVID-19 vaccine once available [15]. Another cross-sectional study from Jordan included 915 adults, and evaluated hesitancy toward the third dose of a COVID-19 vaccine among the general population, with almost half of the participants (56.4%) intending to decline the third dose [16].

Despite studies having been conducted in Jordan addressing willingness to receive a third dose, the willingness amongst HCWs remains unknown [16,17,18]. In addition, there is a paucity of published studies from the Middle East on the uptake rate and the predictors of uptake for a third booster dose of the COVID-19 vaccine among HCWs. As the third dose is not compulsory for HCWs in Jordan, it is essential to assess the willingness of Jordanian HCWs regarding the third dose of the COVID-19 vaccine and to identify barriers to uptake.

## 2. Methodology

### 2.1. Method

This is a cross-sectional study that was conducted to explore the willingness of Jordanian HCWs regarding the third booster dose of the COVID-19 vaccine. An online questionnaire, in English, was developed based on a literature review [19].

#### Study Tool

The questionnaire consisted of three major parts. The participants’ background characteristics, including age, gender, marital status, profession, place of work (public, private, or teaching hospital), nature of work (no contact with COVID-19 patients or contact with COVID-19 patients), and history of COVID-19 infection (no/do not know or yes) were assessed. The HCWs’ willingness to recommend the third dose of the vaccine to their patients was also evaluated using a five-point Likert scale (certainly yes, probably yes, do not know, probably not, and certainly not). The HCWs were then asked about the history of vaccination against seasonal influenza for the winter of 2020/2021, the safety of a vaccine developed in an emergency, trust in science in developing safe and effective new vaccines, and trust in the Ministry of Health to ensure that the third dose of the COVID-19 vaccine was safe. This section also included HCWs’ preferences for acquiring immunity against infectious diseases naturally compared to by vaccination, and if they prefer to wait for more scientific reviews about the safety and efficacy of the third-dose COVID-19 vaccines. HCW booster dose recommendations for patients ≥ 18 years old with chronic diseases and among patients ≥ 65 years old were also evaluated with five-point-scale questions (always, often, sometimes, never, and does not apply to my practice). The list of questions is included in the supplementary material (Survey S1).

### 2.2. Sample and Data Collection

The questionnaire consisted of 19 close-ended questions on “Google Forms”, completed online by the participants. The questionnaire link was distributed through the WhatsApp application between November and December 2021, before the introduction of the third booster dose in Jordan. All the participants were HCWs above 18 years of age and living in Jordan, and whether they had had direct or indirect contact with COVID-19 patients was not considered. Participants who were not Jordanian were excluded from the study.

Four hospitals participated in our study—the King Abdullah University Hospital in the north, the Arab Private Medical Center, the Jordan University Hospital in the middle, and the Al-Karak Governmental Hospital in the south. The four hospitals selected had responded to our invitation sent to all central hospitals with a high number of HCWs and they were representative of the three regions of Jordan. With the permission of the human resource department in each hospital, HCWs were randomly selected and sent a WhatsApp message containing a link to the questionnaire.

### 2.3. Statistical Analysis

The estimated number of physicians in Jordan is 30,000 [20]. A sample size of 270 participants was required for a confidence interval of 90% and a margin of error of 5% [21]. The data were then exported to SPSS (version 26), coded, and analyzed. Descriptive analysis was used to report HCW responses. Descriptive statistics were used to describe the demographic characteristics of the participants. Vaccine uptake proportions were computed using chi-square tests to compare groups’ characteristics and responses with willingness and unwillingness to recommend the third dose of COVID-19 vaccines. The Chi-squared test of independence is a statistical test used to determine whether there is a significant association between two categorical variables. It is often used to analyze the relationship between variables in a contingency table. Statistical significance was set at an alpha of *p* < 0.05.

### 2.4. Ethical Considerations

An ethical review board at Mutah University School of Medicine approved this study (No: 331221) at the beginning of the online questionnaire distribution phase. Data were anonymized, and no personal information was included in the questionnaire.

## 3. Results

Among the total number of 450 WhatsApp messages, 300 were included in the final analysis. Those included physicians, nurses, and pharmacists, with a response rate of 66.7%. This provided a sufficient sample size for a power of 90% 136 with a margin of 5%. The included questionnaires were 196 (65.3%) from physicians, 76 (25.3%) from nurses, and 28 (9.3%) from pharmacists. In total, 81.7% of the participants were younger than 40. The proportions of the participants who were in public, private, and teaching hospitals were 49.0%, 21.3%, and 29.7%, respectively.

The majority (88.0%) of the HCWs had had no contact with COVID-19 patients, and 53.3% of them reported no history of COVID-19 infection (Table 1).

As seen in Table 2, HCWs’ overall willingness in relation to the third dose of the vaccine was 68.4% (49.4% certainly and 19.0% probably), whereas the overall willingness of the HCWs to recommend the third dose to their patients was 73.3% (49.0% certainly and 24.3% probably).

The HCWs’ perceptions regarding administering the third dose of the COVID-19 vaccine to their patients who were ≥18 years old with chronic diseases and to people aged ≥ 65 years old are presented in Table 3. Around one-third of the participants (34.3%) reported that they were “Often” or “Always” unwilling to recommend a third dose to adults aged 65 or older, and a similar proportion (32%) reported the same for their patients with chronic illnesses.

This study identified certain background characteristics associated with the approach regarding the third dose of the COVID-19 vaccine (Table 4). Males were significantly more willing to take the third dose compared to females (82.1% vs. 60.1%, *p*-value < 0.001). Physicians showed higher willingness (75.5%) compared to nurses (51.3%) and pharmacists (64.3%). Unexpectedly, direct contact with COVID-19 patients had no significant effect on HCWs’ willingness rates.

Participants who disagreed with the statement that “*It is preferable to acquire immunity against infectious diseases naturally (by having the disease) than by vaccination”* were more likely to be willing to have the third dose (80.0%) compared to their counterparts who agreed (56.6%) or did not know (57.1%). These differences were statistically significant.

HCWs who agreed with the statement that “*I trust science to develop safe effective new vaccines*” were more likely to be willing to have the third dose (79.1%) compared to their counterparts who reported that they disagreed (27.5%) or did not know (50.0%) (*p*-value <0.001). Participants who reported disagreement with the statement that “*I prefer to wait for more scientific reviews about the safety and efficacy of the Third doseCOVID-19 vaccines*” were more likely to be willing to take the third dose (85.4%) compared to their counterparts who reported that they agreed (58.2%) or did not know (80.5%) (*p*-value < 0.001).

## 4. Discussion

To our knowledge, this is the first study to measure the willingness of Jordanian HCWs regarding the third dose of the COVID-19 vaccine and their intentions to recommend it to their patients. Our results demonstrate that 49.3% of the HCWs showed willingness regarding the third dose of the COVID-19 vaccine. Based on studies published worldwide, the COVID-19 vaccine acceptance rates among HCWs range from 20.0% to 94.0% [22,23,24,25,26,27,28,29,30,31,32]. High rates of 70% and more were reported in studies on HCWs in countries including Germany, France, Poland, Italy [27,33,34,35], Canada [24], Turkey [36], and China [37,38]. The results of this study are consistent with regional figures from Egypt, Palestine, and Qatar, with COVID-19 vaccine hesitancy reaching 50% or more [23,25,26,29,31]. A study from Egypt on HCWs revealed that only 26% of the participants were willing to take the vaccines, whereas 41.9% of participants were reluctant, and the remaining 32.1% refused to take the COVID-19 vaccine. These figures are close to those from Palestine, where only 37.8% (95% CI: 35.0%–40.6%) of HCWs had intentions to get vaccinated. The Palestinian study on HCWs also showed that 31.5% of the participants were undecided, whereas 30.7% of them planned to refuse it. Similar to this study, the willingness of Jordanian HCWs regarding the first dose of the COVID-19 vaccine was 45.6% before it was made locally available [15]. The worst published regional rates were reported in a cohort study on 1704 Iraqi HCWs, with vaccine hesitancy reaching the high rate of 82.1% [39].

A large multicenter study on Arab HCWs revealed that Jordanian HCWs showed only a 21.8% willingness rate [40]. This variation in the willingness towards the COVID-19 vaccines is attributed to sampling bias, obstacles to vaccination, and the different time frames of cross-sectional reports [41,42]. Additionally, the willingness of HCWs regarding COVID-19 vaccines could be attributed to several factors, such as unequal medical backgrounds, different levels of knowledge about the science, and differing awareness of the importance of vaccination [43,44]. Meanwhile, a study conducted among Jordanian HCWs showed 83.3% and 42.6% willingness toward COVID-19 vaccination in general among physicians and nurses, respectively [42]. Our study reported an alarming figure regarding HCWs’ willingness regarding the third dose of the COVID-19 vaccine. This indicates an influence on their perceptions regarding recommending the third dose to their patients or elderly people.

Few demographic data predict willingness regarding the third dose of the COVID-19 vaccine. Males, for example, were more willing than females to get the third dose. This is in accordance with the surveys carried out in the USA, Saudi Arabia, and Ghana [45,46,47]. These figures are not consistent with previous reports, which indicate that females are more likely to comply with preventive measures in response to health threats such as COVID-19 [48,49]. One of the possible explanations is that most females are married. This could render them unwilling to take the vaccine considering its reported effects on breastfeeding and fertility [22,30].

The physicians in this study were more willing to receive the third dose of the COVID-19 vaccine when compared with pharmacists and nurses, similar to the results reported in previous studies [9,27,50,51,52]. For example, results from Greece show that 60–90% of physicians were willing to receive the COVID-19 vaccine, compared to 40–60% of nurses [53]. The positive attitudes of physicians towards COVID-19 vaccines are highly significant because of these individuals’ anticipated risks of infection with COVID-19, and direct care of patients was also found to increase the probability of COVID-19 vaccine uptake [52,54,55]. Therefore, these individuals might alter their patients’ attitudes toward the vaccine. During the pandemic, special hospitals were allocated for the management of COVID-19; subgroup analysis showed no differences between those with direct contact and those without direct contact.

HCWs from the private health sector were more willing to accept the third dose of the COVID-19 vaccine compared to public health sector workers. This is consistent with figures from Cyprus and opposite to those from Ghana [40,56]. This might be explained by differences in education levels and follow-up systems at private hospitals [56].

Vaccine hesitancy appears to be multifactorial. The most commonly reported barriers to third-dose COVID-19 uptake are concerns regarding vaccine safety and efficacy, distrust in the science and health systems, and anxiety regarding the rapid development of the vaccine [33,34,40]. Worries about vaccine safety are one of the key identified barriers to vaccine uptake, as reported by around two-thirds of the participants [57]. The results from Egypt on HCWs showed that higher income and increased years of work experience are positive predictors of the willingness to receive a vaccine. Finally, a study from Palestine identified several predictors of COVID-19 vaccine uptake that are consistent with our results. Higher levels of intention were reported among males (OR: 2.7, 95%CI: 2.0–3.7), younger age groups (OR: 1.7, 95%CI: 1.1–2.8), physicians (OR: 2.9, 95%CI: 2.0–4.0), HCWs in non-governmental settings (OR: 1.4, 95%CI: 1.1–1.9), those who had previously received the influenza vaccine (OR: 4.0, 95%CI: 2.3–7.1), and those who had greater COVID-19-related knowledge (OR: 1.7, 95%CI: 2.3–7.1).

Another important outcome of this study is that HCWs trusted the Ministry of Health to ensure that the dose of the COVID-19 vaccine was safe. Additionally, HCWs trusted the science for the development of safe and effective new vaccines and were disinclined to wait for more scientific reviews about the safety and efficacy of the third vaccine. In contrast, data from France, Belgium, and Canada showed distrust in the health authorities to ensure vaccine safety [19].

The main limitation of our study is that we reached participants through WhatsApp messages and did not receive the details of the non-respondents. Dentists were difficult to reach, and therefore, we excluded them from the study. Another point we discovered during the analysis was that the vaccine type received by the HCW could not be linked to their willingness to recommend any vaccine. This point was also applicable when we considered HCWs’ knowledge about the reported efficacy of some of the COVID-19 vaccines versus others. These points should be studied in detail in future studies.

Additionally, the fieldwork was conducted near the end of 2021, when the Omicron variant was dominant locally and internationally. This might have affected the results, although the participants were asked about their overall experiences with the third dose. Another limitation is that we excluded non-Jordanian HCWs. Many non-Jordanian healthcare workers work on a temporary basis; therefore, we decided to exclude this important group.

## 5. Conclusions

Most of the published studies have covered HCWs’ vaccine uptake and predictors of uptake. Few published studies have assessed the uptake rate for the third dose amongst HCWs. This study revealed that HCWs in Jordan show a limited willingness to take the third COVID-19 vaccine. This study identified selected barriers to the uptake of the third dose. Additionally, third-dose vaccine hesitancy has a negative impact on HCWs’ likeliness to promote the third dose for their patients and the community overall. Different approaches can be adopted by the government to combat vaccine hesitancy among HCWs. This should be the focus of health-promotion programs in Jordan, particularly those directed toward HCWs.

## Figures and Tables

**Table 1:** Distributions of study participants by background characteristics.

Characteristics	Participants Group	Number (%)
Age (years)	Less than 40	245 (*81.7*)
	40 to 50	42 (*14.0*)
	50 or more	13 (*4.3*)
	Total	300 (*100*)
Gender	Female	188 (*62.7*)
	Male	112 (*37.3*)
	Total	300 (*100*)
Marital status	Married	166 (*55.3*)
	Single	134 (*44.7*)
	Total	300 (*100*)
Profession	Physicians	196 (*65.3*)
	Nurse	76 (*25.3*)
	Pharmacists	28 (*9.3*)
	Total	300 (*100*)
Place of work	Public sector	147 (*49.0*)
	Private sector	64 (*21.3*)
	University/teaching hospital	89 (*29.7*)
	Total	300 (*100*)
Nature of work	No contact with COVID patients	265 (*88.3*)
	Contact with COVID patients	35 (*11.7*)
	Total	300 (*100*)
History of COVID infection	No	160 (*53.3*)
	Yes	140 (*46.7*)
	Total	300 (*100*)

**Table 2:** The overall willingness regarding the third dose of the COVID-19 vaccine.

**If third-dose COVID-19 vaccines were available at your workplace,** **would you be willing to recommend them to your patients?**	**Number (%)**
Yes, certainly	147 (*49.0*)
Yes, probably	73 (*24.3*)
Do not know	32 (*10.7*)
No, probably not	29 (*9.7*)
No, certainly not	19 (*6.3*)
Total	300 (*100.0*)
**If third-dose COVID-19 vaccines were available at your workplace,** **would you be willing to be vaccinated yourself?**	**Number (%)**
Yes, certainly	148 (*49.4*)
Yes, probably	57 (*19.0*)
Do not know	19 (*6.3*)
No, probably not	43 (*14.3*)
No, certainly not	33 (*11.0*)
Total	300 (*100.0*)

**Table 3:** HCWs’ perceptions regarding administering third doses to their patients.

Item		Number (%)
Are you sometimes unwilling to recommend the third dose of the COVID-19 vaccine to your patients, for example, when you have questions about the benefits or risks? (Among adults ≥18 years old with chronic diseases)	Never	93 (*31.0*)
	Sometimes	80 (*26.7*)
	Often	54 (*18.0*)
	Always	42 (*14.0*)
	Does not apply to my practice	31 (*10.3*)
	Total	300 (*100*)
Are you sometimes unwilling to recommend the third dose of the COVID-19 vaccine to your patients, for example, when you have questions about the benefits or risks? (Among adults aged ≥65 old)	Never	84 (*28.0*)
	Sometimes	81 (*27.0*)
	Often	52 (*17.3*)
	Always	51 (*17.0*)
	Does not apply to my practice	32 (*10.7*)
	Total	300 (*100*)

**Table 4:** Comparison of HCWs’ characteristics and responses with willingness and unwillingness to recommend the third dose of COVID-19 vaccines.

		Unwillingness Frequency (%)	Willingness Frequency (%)	Total	*p*-Value
	Overall	95 (31.7)	205 (68.3)	300	*0.491*
**Age (years)**	Less than 40	81 (*33.1*)	164 (*66.9)*	245	
	40 to 50	10 (*23.8*)	32 (*76.2)*	42	
	50 or more	4 (*30.8*)	9 (*69.2)*	13	
	Total	95 (*31.7*)	205 (*68.3)*	300	
**Gender**	Female	75 (*39.9*)	113 (*60.1*)	188	*<0.001*
	Male	20 (*17.9*)	92 (*82.1*)	112	
	Total	95 (*31.7*)	205 (*68.3*)	300	
**Marital status**	Married	50 (*30.1*)	116 (*69.9*)	166	*0.522*
	Single	45 (*33.6*)	89 (*66.4*)	134	
	Total	95 (*31.7*)	205 (*68.3*)	300	
**Profession**	Physicians	48 (*24.5*)	148 (*75.5*)	196	*<0.001*
	Nurse	37 (*48.7*)	39 (*51.3*)	76	
	Pharmacists	10 (*35.7*)	18 (*64.3*)	28	
	Total	95 (*31.7*)	205 (*68.3*)	300	
**Place of work**	Public sector	56 (*38.1*)	91 (*61.9*)	147	*0.011*
	Private sector	11 (*17.2*)	53 (*82.8*)	64	
	University	28 (*31.5*)	61 (*68.5*)	89	
	Total	95 (*31.7*)	205 (*68.3*)	300	
**Nature of work (contact with COVID-19 patients)**	No	83 (*31.3*)	182 (*68.7*)	265	*0.723*
	Yes	12 (*34.3*)	23 (*65.7*)	35	
	Total	95 (*31.7*)	205 (*68.3*)	300	
**History of COVID-19 infection**	No	48 (*30.0*)	112 (*70.0*)	160	*0.507*
	Yes	47 (*33.6*)	93 (*66.4*)	140	
	Total	95 (*31.7*)	205 (*68.3*)	300	
**Received Influenza vaccine** **for winter 2020/2021**	Do not know	4 (*33.3*)	8 (*66.7*)	12	*0.182*
	No	69 (*35.0*)	128 (*65.0*)	197	
	Yes	22 (*24.2*)	69 (*75.8*)	91	
	Total	95 (*31.7*)	205 (*68.3*)	300	
**The safety of a vaccine developed in an emergency cannot be considered guaranteed**	Agree	56 (*36.8*)	96 (*63.2*)	152	*0.145*
	Disagree	17 (*25.4*)	50 (*74.6*)	67	
	Do not know	22 (*27.2*)	59 (*72.8*)	81	
	Total	95 (*31.7*)	205 (*68.3*)	300	
**It is preferable to acquire immunity naturally than by vaccination**	Agree	53 (*43.4*)	69 (*56.6*)	122	*<0.001*
	Disagree	30 (*20.0*)	120 (*80.0*)	150	
	Do not know	12 (*42.9*)	16 (*57.1*)	28	
	Total	95 (*31.7*)	205 (*68.3*)	300	
**I trust science to develop safe and effective new vaccines**	Agree	46 (*20.9*)	174 (*79.1*)	220	*<0.001*
	Disagree	29 (*72.5*)	11 (*27.5*)	40	
	Do not know	20 (*50.0*)	20 (*50.0*)	40	
	Total	95 (*31.7*)	205 (*68.3*)	300	
**I trust the ministry of health to ensure that the third dose is safe**	Agree	24 (*13.6*)	152 (*86.4*)	176	*<0.001*
	Disagree	41 (*63.1*)	24 (*36.9*)	65	
	Do not know	30 (*50.8*)	29 (*49.2*)	59	
	Total	95 (*31.7*)	205 (*68.3*)	300	
**I prefer to wait for more scientific reviews about safety and efficacy of the third dose**	Agree	76 (*41.8*)	106 (*58.2*)	182	*<0.001*
	Disagree	12 (*14.6*)	70 (*85.4*)	82	
	Do not know	7 (*19.4*)	29 (*80.6*)	36	
	Total	95 (*31.7*)	205 (*68.3*)	300	

## Data Availability

The data and materials used during the current study are available from the corresponding author upon reasonable request.

## Supplementary Materials

The following supporting information can be downloaded at: https://www.mdpi.com/article/10.3390/idr15020022/s1, Survey S1. Healthcare workers attitude towards Third dose COVID-19 vaccines in Jordan.
