# Supplementary material for: Willingness of Healthcare Workers to Recommend or Receive a Third COVID-19 Vaccine Dose: A Cross-Sectional Study from Jordan
Source: Infect Dis Rep. 2023 Apr 6;15(2):210–21. doi: 10.3390/idr15020022 (PMC10138052; doi:10.3390/idr15020022)
Supplement: Supplementary file 1 [file idr-15-00022-s001.zip › idr-2261084-supplementary.pdf]

Healthcare workers attitude towards Third dose COVID-19 vaccines in Jordan

The results of this survey will provide us insights in how the healthcare workers interact with COVID-19 vaccines especially the third dose.But we will not spam you in any way!So do not worry,you will remain completely anonymous.

*Required

1. Age *

____

2. Gender *

Mark only one oval.

- Male
- Female

3. What is your current marital status? *

Mark only one oval.

- ◯ single
- ◯ married without children
- ◯ married with children

4. What is your profession in the healthcare field? *

Mark only one oval.

- medical doctor-surgeon
- medical doctor-internal medicine
- medical doctor-pediatrics
- medical doctor-OB/GYN
- medical doctor-family medicine
- medical doctor-anesthesiologist
- medical doctor-lab medicine
- medical doctor-general practice
- medical doctor-other
- medical student
- nurse-practicing
- nurse-academic
- nursing student
- dentist
- dental student
- pharmacist
- pharmacy student
- research-principal investigator
- research-post doctorate
- research-PhD student
- nutritionist
- physiotherapist

5. place of work *

Mark only one oval.

- university hospitaly
- Royal Medical Services
- MOH
- private sector

6. Location of work? *

Mark only one oval.

- Irbid
- Mafraq
- Zarqa
- Ajloun
- Jerash
- Amman
- Madaba
- Balqa
- Karak
- Tafileh
- Maan
- Aqaba

7. Nature of work: *

Mark only one oval.

- ⬭ Work clinically,without direct contact with patients
- ⬭ work clinically,with direct contact with patients
- ⬭ work clinically,with direct contact with COVID-19 patients
- ⬭ No clinical work

8. history of COVID-19 infection: *

Mark only one oval.

- ⬭ yes
- ⬭ no
- ⬭ do not know

Healthcare workers attitude towards Third dose COVID-19 vaccines in Jordan

The results of this survey will provide us insights in how the healthcare workers interact with COVID-19 vaccines especially the third dose.But we will not spam you in any way!So do not worry,you will remain completely anonymous.

9. Age *

10. Gender *

Mark only one oval.

- Male
- Female

11. What is your current marital status? *

Mark only one oval.

- single
- married without children
- married with children

12. What is your profession in the healthcare field? *

Mark only one oval.

- medical doctor-surgeon
- medical doctor-internal medicine
- medical doctor-pediatrics
- medical doctor-OB/GYN
- medical doctor-family medicine
- medical doctor-anesthesiologist
- medical doctor-lab medicine
- medical doctor-general practice
- medical doctor-other
- medical student
- nurse-practicing
- nurse-academic
- nursing student
- dentist
- dental student
- pharmacist
- pharmacy student
- research-principal investigator
- research-post doctorate
- research-PhD student
- nutritionist
- physiotherapist

13. place of work *

Mark only one oval.

- university hospitaly
- Royal Medical Services
- MOH
- private sector

14. Location of work? *

Mark only one oval.

- Irbid
- Mafraq
- Zarqa
- Ajloun
- Jerash
- Amman
- Madaba
- Balqa
- Karak
- Tafileh
- Maan
- Aqaba

15. Nature of work: *

Mark only one oval.

- Work clinically,without direct contact with patients
- work clinically,with direct contact with patients
- work clinically,with direct contact with COVID-19 patients
- No clinical work

16. history of COVID-19 infection: *

Mark only one oval.

- yes
- no
- do not know

Healthcare workers attitude towards COVID-19 vaccines in Jordan

17. If Third dose COVID-19 vaccines were available at your work place, would you be willing to recommend it to your patients? *

Mark only one oval.

- yes,certainly
- yes,probably
- No,probably not
- No,certainly not
- Do not know

18. If Third dose COVID-19 vaccines were available at your work place, would you be willing to be vaccinated yourself? *

Mark only one oval.

- yes,certainly
- yes,probably
- No,probably not
- No,certainly not
- Do not know

19. How much do you rate your Third dose COVID-19 vaccine acceptance? *

Mark only one oval.

- ( ) High
- ( ) Moderate
- ( ) Hesitancy/reluctance

20. Were you vaccinated against seasonal influenza for the winter 2020/21 season? *

Mark only one oval.

- ( ) Yes
- ( ) No
- ( ) Do not know

21. Do you sometimes hesitate to recommend boaster dose of vaccines for your patients, for example, when you have questions about their benefits or risks? *

Mark only one oval per row.

	Never	sometimes	often	alw
**For adults (≥ 18 years old) with a chronic diseas**				
**Among adults aged ≥ 65 years old**				

22. The safety of a vaccine developed in an emergency, during an epidemic, cannot be considered guaranteed *

Mark only one oval.

- Agree
- Disagree
- Do not know

23. In your opinion, for the population as a whole, how serious is COVID-19 on a scale of 0 to 10? *

Mark only one oval.

	low
0	⬭
1	⬭
2	⬭
3	⬭
4	⬭
5	⬭
6	⬭
7	⬭
8	⬭
9	⬭
10	⬭
	high

24. It is preferable to acquire immunity against infectious diseases naturally (by having the disease) than by vaccination *

Mark only one oval.

- Agree
- Disagree
- Do not know

25. I trust science to develop safe effective new vaccines *

Mark only one oval.

- Agree
- Disagree
- Do not know

26. I trust the ministry of health to ensure that the boaster dose of COVID-19 vaccines are safe *

Mark only one oval.

- Agree
- Disagree
- Do not know

27. I prefer to wait for more scientific reviews about the safety and efficacy of the Third dose COVID-19 vaccines *

Mark only one oval.

- Agree
- Disagree
- Do not know

This content is neither created nor endorsed by Google.

Google Forms
